# Evidence of active whole-genome doubling (WGD) subclones in non-WGD tumors

**DOI:** 10.1126/sciadv.aee7939

**Published:** 2026-10-30

**Authors:** Funan He, Mehwish Noureen, Eloise Dray, Myron S. Ignatius, Xiaojing Wang, Siyuan Zheng

**Affiliations:** ^1^ Greehey Children’s Cancer Research Institute, UT Health San Antonio, San Antonio, TX, USA.; ^2^ Department of Population Health Sciences, UT Health San Antonio, San Antonio, TX, USA.; ^3^ Department of Biochemistry and Structural Biology, UT Health San Antonio, San Antonio, TX, USA.; ^4^ Mays Cancer Center at UT Health San Antonio, San Antonio, TX, USA.; ^5^ Department of Molecular Medicine, UT Health San Antonio, San Antonio, TX, USA.; ^6^ Barshop Institute for Longevity and Aging Studies, UT Health San Antonio, San Antonio, TX, USA.

## Abstract

Whole-genome doubling (WGD) occurs frequently in human cancer and influences tumor evolution, metastasis, and treatment response. Here, we integrate bulk, single-cell, and spatial data to dissect the subclonal dynamics of WGD. Single-cell data analysis shows that WGD cells are prevalent even in tumors that are classified as non-WGD (nWGD). WGD subclones actively acquire somatic copy number alterations as they expand, suggesting ongoing evolution. We developed and rigorously validated a gene expression–based method, WAGES, to map WGD cell clonal fraction in bulk tumors. Applying WAGES to nWGD tumors, we reveal genomic correlates including *TP53* mutations and chromosomal instability that are consistent with the presence of WGD subclones. Clinically, WAGES scores are strongly correlated with tumor metastatic potential, response to treatments including chemotherapy and immunotherapy, and patient prognosis. These findings reveal widespread WGD subclones in nWGD tumors and demonstrate their substantial impact on cancer evolution and therapeutic outcomes.

## INTRODUCTION

Whole-genome doubling (WGD) occurs frequently in human cancer (*1*, *2*). Studies in model organisms show that WGD increases genetic diversity and accelerates evolution (*3*–*5*). In cancer, WGD exerts pleiotropic effects on cancer cells by buffering deleterious mutations (*6*), propagating chromosomal instability (*7*–*9*), altering chromatin structure (*10*), and mediating immune evasion (*11*, *12*). Clinically, WGD is associated with cancer metastasis (*13*–*15*), recurrence (*16*, *17*), patient prognosis (*7*, *15*), and ancestry (*18*).

WGD is thought to arise from mitotic errors (*15*, *19*, *20*). The rapid proliferation of cancer cells creates ample opportunities for WGD, and laboratory observations confirm common presence of WGD cells in cell culture (*21*). In patient tumors where space and nutrients are limited, newly emerged WGD cells face competition from isogenic cells that did not undergo WGD (nWGD cells) (*22*). The dynamics of this competition determine whether the nascent WGD clone remains marginal or evolves into a fully developed tumor. Despite previous reports on WGD subclones (*6*, *12*, *23*, *24*), WGD is still largely considered a binary, tumor-level event. This perception obscures the potential presence of WGD as a subclonal state and limits insight into its evolutionary dynamics. Delineation of these dynamics is crucial for understanding the evolutionary trajectory of WGD tumors.

Here, we integrate multimodal datasets to investigate WGD/nWGD cell dynamics in human cancer. We leverage single-cell DNA sequencing (scDNA-seq) to examine the cellular prevalence and genomic evolution of WGD states, and we develop a gene expression–based method, WGD-associated gene expression signature (WAGES), to estimate the fraction of WGD cells within bulk tumor samples. Our study provides evidence that WGD frequently exists as subclones in tumors classified as nWGD and that these subclones have clinically important consequences.

## RESULTS

### Coexistence of WGD and nWGD cells in nWGD tumors

To investigate WGD in single cells, we used a scDNA-seq dataset published by Funnell *et al.* (*25*). The dataset comprised whole-genome sequences of more than 55,000 cells from 26 ovarian and breast cancer samples and eight control cell lines. In this study, we excluded the eight control cell lines, which were 184-hTERT and its *TP53*/*BRCA1*/*BRCA2*-edited derivatives. From the allele-specific absolute copy number profile, we observed a clear bimodal distribution of the fraction of major alleles with two or higher copies in single cells (Fig. 1A). The pattern was previously used to classify WGD status for bulk samples (*15*, *26*). Accordingly, we identified 27,484 WGD cells and 12,550 nWGD cells (Materials and Methods). The median ploidy of WGD cells was 2.8, compared with 1.8 for nWGD cells (fig. S1A). These ploidy values were consistent with those of matched bulk patient tumors (fig. S1B). Most WGD cells underwent one doubling except in two ovarian tumors where a subset of cells apparently underwent two doublings (fig. S1C). For simplicity, we excluded cells that underwent two doublings from subsequent analyses because they may have a different evolutionary history. Their exclusion did not affect WGD clonal fraction in the corresponding tumors.

**Fig. 1.**
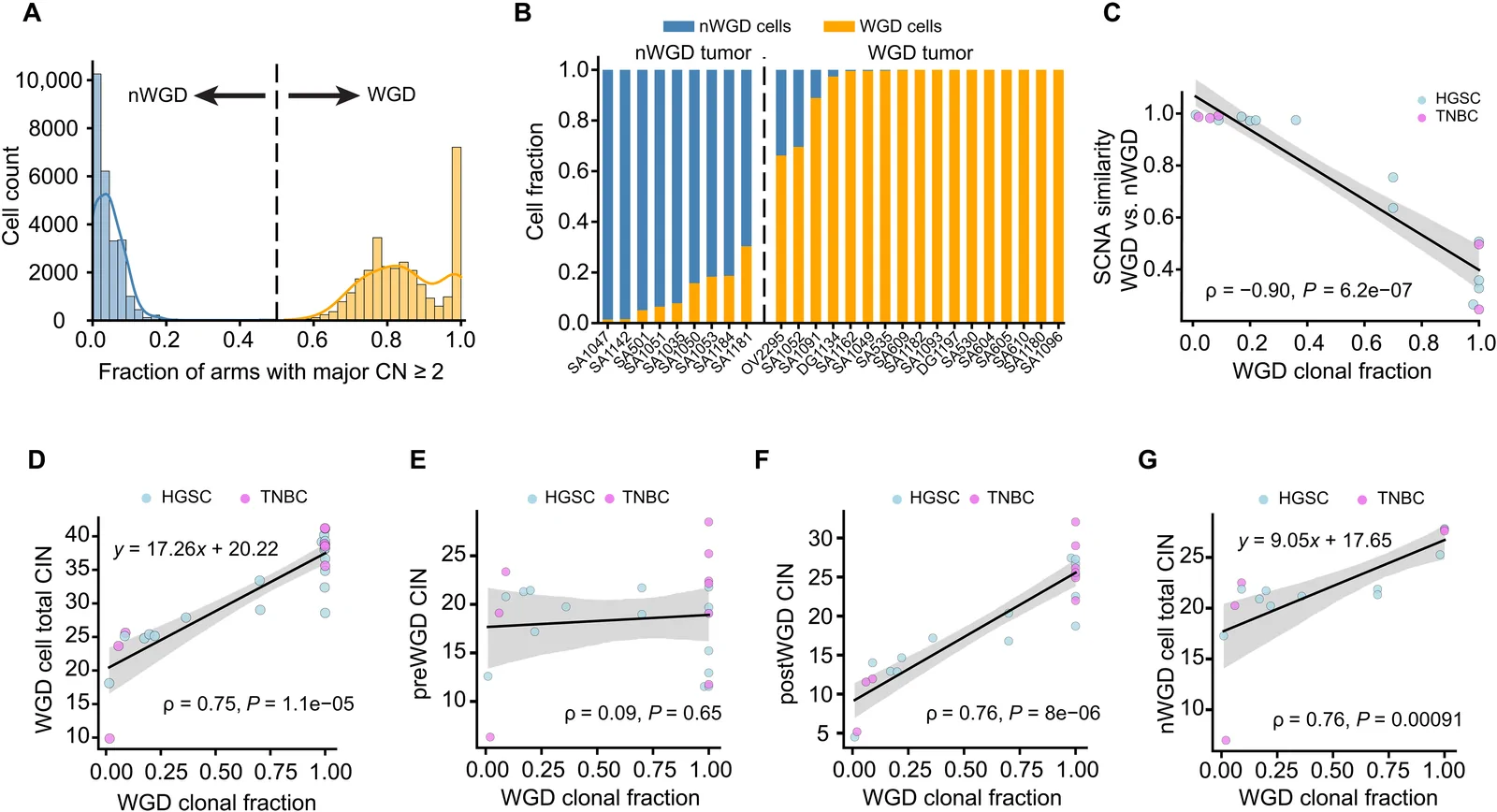
WGD cells are prevalent in cancer and are associated with copy number evolution. (**A**) Fraction of chromosome arms with major allele copy number ≥2 follows a bimodal distribution. (**B**) Proportions of WGD and nWGD cells across cancer samples. Samples are ordered by WGD clonal fraction, i.e., proportions of WGD cells. Tumor WGD status is categorized by WGD clonal fraction (cutoff of 0.5). (**C** to **G**) Correlation between WGD clonal fraction and copy number similarity between WGD and nWGD cells (C), CIN scores of WGD cells (D), preWGD CIN scores of WGD cells (E), postWGD CIN scores of WGD cells (F), and CIN scores of nWGD cells (G). HGSC, high-grade serous ovarian cancer; TNBC, triple-negative breast cancer. Each dot represents one sample, and the color denotes cancer type. Correlation and *P* values are calculated using Spearman correlation. Slope and intercept are calculated using linear least-squares regression. Shading represents 95% confidence interval.

We classified a tumor as WGD if WGD cells constituted the majority (>50%), or nWGD if otherwise. Regardless of the tumor’s overall WGD status, WGD cells were observed in all 26 tumor samples (Fig. 1B). On average, 95% of cancer cells in WGD tumors were WGD cells, and most WGD tumors (14 of 17, 82%) predominantly comprised WGD cells (proportion, >97%). Unexpectedly, 12% of cancer cells in nWGD tumors were WGD cells (range, 1.4 to 30%). These results show pervasive presence of WGD cells in tumors and that coexistence of WGD and nWGD cells was common in nWGD tumors.

### Correlation of WGD clonal fraction with SCNA indicates ongoing evolution

WGD cells arise from an ancestral nWGD cell through mitotic errors (*19*). In a WGD/nWGD mixed tumor where WGD and nWGD cells compete for space and resources (*12*, *22*), WGD clonal fraction, defined as the proportion of WGD cells, represents a snapshot of this evolutionary competition.

We found that WGD clonal fraction was negatively correlated with copy number similarities between WGD and nWGD cells from the same tumor (ρ = −0.90, *P* = 6.2 × 10^−7^, Spearman correlation; Fig. 1C). This result suggests that, at low WGD clonal fractions, WGD cells largely retain the somatic copy number alteration (SCNA) profile of the ancestral nWGD cells, likely due to recent divergence. As WGD clones expand, both WGD and nWGD cells accumulate SCNAs, leading to greater differences.

To further test this, we calculated a chromosomal instability (CIN) score for each cell to quantitatively aggregate the impact of SCNAs on the genome (Materials and Methods). WGD cells exhibited significantly higher CIN scores than nWGD cells (fig. S2A), and, within WGD cells, WGD clonal fraction was positively correlated with CIN scores (ρ = 0.75, *P* = 1.1 × 10^−5^, Spearman correlation; Fig. 1D). To understand whether this correlation reflected ongoing genomic evolution, we timed SCNAs in WGD cells into those occurring before WGD (preWGD) and those occurring after WGD (postWGD) (Materials and Methods). We observed that WGD clonal fraction was correlated with postWGD, but not preWGD, SCNAs (Fig. 1, E and F, and fig. S2B), consistent with continuous acquisition of SCNAs as WGD cell populations expand.

Within nWGD cells, CIN scores were also positively correlated with WGD clonal fraction (ρ = 0.76, *P* = 9.1 × 10^−4^, Spearman correlation; Fig. 1G), suggesting that nWGD cells continue to acquire SCNAs as well. Notably, CIN scores increased at a rate nearly twice as high in WGD cells than in nWGD cells (regression slope, 17 versus 9; Fig. 1, D and G). The difference remained after normalizing CIN scores by cell ploidy (fig. S2C), suggesting that WGD cells have inherently more unstable genomes than nWGD cells.

Together, our data reveal that WGD cells are more prevalent in nWGD tumors than previously appreciated. The positive correlation between WGD clonal fraction and CIN scores indicates that these subclones experience ongoing chromosomal instability that can potentially enhance their evolutionary adaptability under selective pressures.

### WGD-associated gene expression signature

Sample sizes in scDNA-seq datasets are often modest. To generalize our observation that WGD cells were common in nWGD tumors, we developed a WGD-specific gene signature and used it to analyze larger bulk tumor datasets. Briefly, using data from TCGA and PCAWG, we constructed multivariable linear regression models to associate gene expression with tumor WGD status while controlling for potential confounders including tumor purity and disease stage. We adjusted for variations caused by unobserved clinical and molecular factors in the model (Materials and Methods).

The resulting signature, termed “WGD-associated gene expression signature (WAGES),” comprised 598 genes (table S1). Functionally, WAGES genes were enriched in RNA metabolism and processing, cell cycle regulation, and p53 pathway (fig. S3A). To verify WAGES, we applied it to the TCGA dataset (table S2). As anticipated, WGD tumors showed significantly higher WAGES scores than nWGD tumors. Although samples that underwent two genome doublings were not used in WAGES construction, they showed higher scores than nWGD samples and samples that underwent one doubling (Fig. 2A). Across cancer types, WAGES scores were positively correlated with WGD frequency (ρ = 0.77, *P* = 1.9 × 10^−7^, Spearman correlation; fig. S3B).

**Fig. 2.**
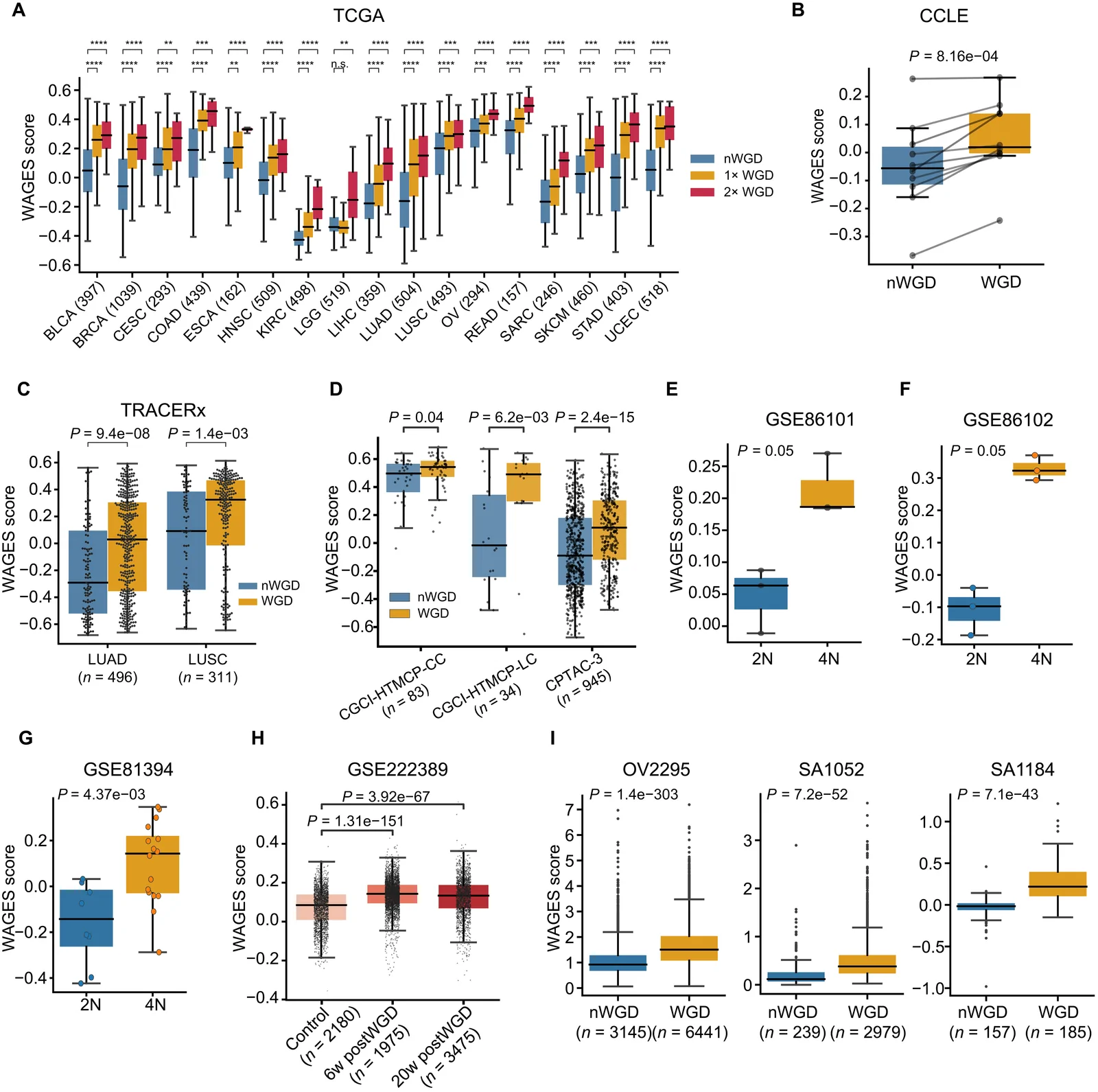
Validation of WAGES. (**A**) WAGES scores of TCGA tumors. Seventeen cancer types with sufficient WGD (*n* ≥ 20) and nWGD samples (*n* ≥ 20) are included. Sample size is given in the *x*-axis label for each cohort. The boxes and middle lines within represent the interquartile range (IQR) and median. The top and bottom whiskers represent values within 1.5× IQR of the upper and lower quartiles, respectively. Box plots in other panels are interpreted similarly. *P* values are calculated using one-sided Wilcoxon rank sum test (n.s., not significant; **P ≤ 0.01; ***P ≤ 0.001; ****P ≤ 0.0001). (**B**) WAGES scores of cancer cell lines from CCLE. Each dot represents the average score of a cancer type. WGD and nWGD cell lines from the same cancer types are connected with a line. *P* value is calculated using paired *t* test. (**C**) WAGES scores of TRACERx lung samples. *P* value is calculated using one-sided Wilcoxon rank sum test. (**D**) WAGES scores of tumor samples from three additional datasets. Each dot represents a tumor sample. *P* value is calculated using one-sided Wilcoxon rank sum test. (**E** to **G**) WAGES scores of experimentally sorted 2N and 4N clones. Each dot represents a sample. *P* value is calculated using one-sided Wilcoxon rank sum test. (**H**) WAGES scores of isogenic 2N and 4N *TP53*^−/−^ RPE1 cells using scRNA-seq data. Each dot represents a cell. *P* value is calculated using one-sided Wilcoxon rank sum test. 6w, 6 weeks; 20w, 20 weeks. (**I**) WAGES scores of WGD and nWGD tumor cells using scRNA-seq data. Each dot represents a cell. *P* value is calculated using one-sided Wilcoxon rank sum test.

To validate WAGES, we applied it to cell lines and bulk cancer samples. In Cancer Cell Line Encyclopedia (CCLE), WGD cell lines showed higher WAGES scores than nWGD cell lines of the same histology (*P* = 8.2 × 10^−4^, paired *t* test; Fig. 2B). In TRACERx lung data, WGD cancer samples also showed higher scores than nWGD samples (Fig. 2C). The pattern was replicated in three additional solid tumor datasets comprising various cancer types (*n* = 1062; Fig. 2D). We then applied the signature to experimentally sorted, isogenic diploid (2N) and tetraploid (4N) RPE1 cells (*27*). Similarly, 4N samples showed higher scores than 2N samples (*P* = 0.05, Wilcoxon rank sum test; Fig. 2, E and F), and the pattern remained regardless of the methods used to induce WGD (fig. S3, C and D). Last, we applied the signature to a dataset consisting of isogenic 2N and 4N colorectal cancer cell line DLD-1 (*28*). 4N samples again showed higher scores than 2N samples (*P* = 4.4 × 10^−3^, Wilcoxon rank sum test; Fig. 2G).

We then tested WAGES on single-cell RNA sequencing (scRNA-seq) datasets. The first test dataset comprised 2N *TP53*^−/−^ RPE1 cells and isogenic 4N cells collected at 6 and 20 weeks after WGD induction (*10*). Both 6-week and 20-week WGD cells showed significantly higher WAGES scores than the parental nWGD cells (*P* < 0.001, Wilcoxon rank sum test; Fig. 2H). Next, we leveraged the Funnell dataset (*25*) where scDNA-seq and scRNA-seq were generated for 19 cancer samples. Of them, six were admixtures with relatively high proportions of WGD and nWGD cells (WGD cell proportion, 10 to 90%). We were able to confidently assign WGD status to cells in the scRNA-seq data in three of the six samples based on the relative copy number profiles (Materials and Methods; fig. S4, A to C). Cells identified as WGD expressed more genes and higher transcriptomic counts in scRNA-seq (fig. S4, D and E), concordant with previous finding that polyploid cells express higher total mRNA load (*29*). Similar to other validation datasets, we observed higher WAGES scores in WGD cells (*P* < 0.001, Wilcoxon rank sum test; Fig. 2I). Together, these results provide validation for WAGES.

### WAGES measures WGD clonal fraction in mixed samples

Validation of WAGES in single cells suggests that it can potentially quantify WGD clonal fraction in WGD/nWGD mixed tumors. To formally test this, we first examined samples of the same patient origin with varying WGD clonal fractions in the Funnell dataset. We observed consistently higher WAGES scores in samples that exhibited higher WGD clonal fractions (Fig. 3, A and B, and fig. S5, A and B). Across samples, WAGES scores calculated from pseudobulk expression data correlated with WGD clonal fractions estimated from scDNA-seq data (ρ = 0.77, *P* = 0.072, Spearman correlation; Fig. 3C).

**Fig. 3.**
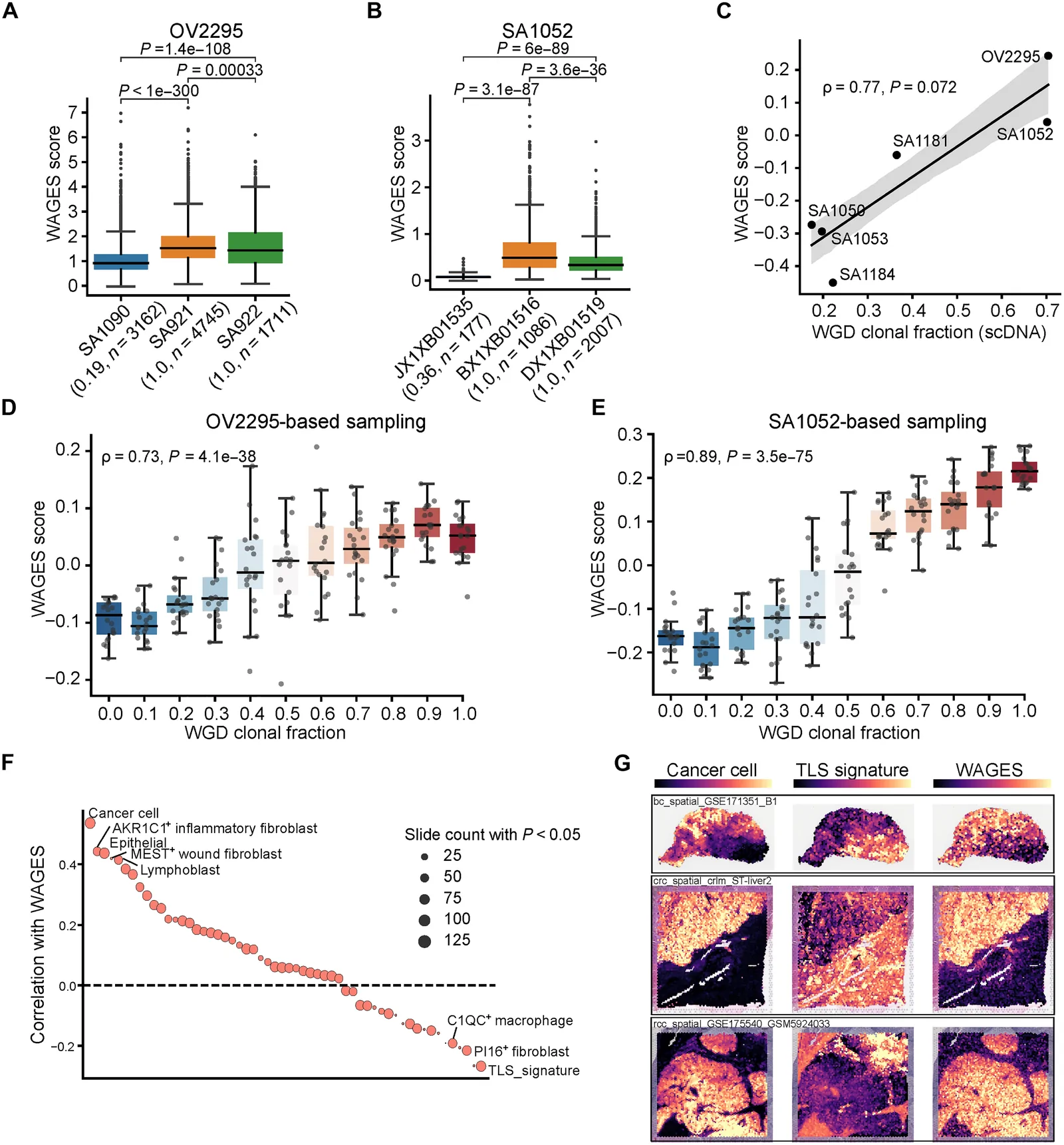
WAGES score measures WGD clonal fraction. (**A** and **B**) WAGES scores increase with WGD clonal fraction across samples from the same patient. Each dot represents a cell. The numbers in parentheses on the *x* axis show WGD clonal fraction (estimated from scDNA data) and number of cells (calculated from scRNA data). The boxes and middle lines within represent the IQR and median. The top and bottom whiskers represent values within 1.5× IQR of the upper and lower quartiles, respectively. Box plots in other panels are interpreted similarly. *P* values are calculated using two-sided Wilcoxon rank sum test. (**C**) Correlation between WAGES scores calculated with pseudobulk expression data (*y* axis) and WGD clonal fraction from scDNA-seq data (*x* axis). Only samples with WGD clonal fraction between 0.1 and 0.9 are included. Each dot represents a sample. ρ and *P* values are calculated using Spearman correlation. (**D** and **E**) Correlation between WAGES scores and WGD clonal fraction in synthetic data. Each dot represents one synthetic sample generated at the specific WGD clonal fraction. ρ and *P* values are calculated using Spearman correlation. (**F**) Correlation between the WAGES signature and other cell signatures in the spatial transcriptomic dataset. Each dot represents the average correlation across the 137 slides. The size of the dot represents the number of slides with a significant *P* value (<0.05). *y* axis, the average correlation coefficient. Correlation coefficient and *P* values are calculated using Pearson correlation. (**G**) Spatial slides showing colocalization of WAGES with cancer cell signature, and mutual exclusivity with tertiary lymphoid structure (TLS).

In more controlled computational experiments, we constructed pseudobulk samples by mixing WGD and nWGD cells at a series of WGD clonal fractions. At each WGD clonal fraction, we randomly selected WGD and nWGD cells from a tumor sample to construct the pseudobulk (Materials and Methods). WAGES scores of the pseudobulk samples were significantly correlated with their WGD clonal fractions (*P* < 0.001, Spearman correlation; Fig. 3, D and E), indicating that the WAGES score can reliably reflect WGD clonal fraction.

Another factor that can affect WAGES scores is tumor impurity caused by nontumor cells. Presence of nontumor cells may dilute or even skew signature scores if they also strongly express the signature. In TCGA and TRACERx datasets, we observed very low WAGES scores in normal samples compared with tumors (fig. S5, C and D), suggesting that the signature was more specific to cancer cells. To further test this, we applied WAGES to a pan-cancer spatial transcriptomic dataset that consisted of 137 tumor samples (*30*). Colocalization analysis showed that WAGES scores highly colocalized with cancer cells (Fig. 3, F and G). Specifically, significant positive spotwise correlation between WAGES scores and the cancer cell signature was observed in 97.8% of the cohort (fig. S5E). The colocalization of WAGES and cancer cells was replicated in other independent spatial transcriptomic data (fig. S5G). Significant negative correlation was observed in 89.8% of the cohort between WAGES and tertiary lymphoid structure, an organized immune cell aggregate that can influence cancer patient responses to immunotherapy (Fig. 3, F and G, and fig. S5F) (*31*). Together, these data show that the WAGES score can reflect WGD clonal fraction and is cancer cell specific.

### WAGES reveals genomic evidence of WGD clones in nWGD tumors

Next, we used WAGES as a proxy for WGD clonal fraction to interrogate bulk tumor samples. We focused on nWGD tumors because WGD tumors predominantly consisted of WGD cells as shown by scDNA-seq data (Fig. 1B) and multiregional sequencing (*6*). We posited that nWGD tumors with higher WAGES scores are more likely to harbor WGD subclones.

The WAGES score was significantly correlated with tumor CIN scores in all 16 cancer types tested (FDR of <0.05, Fig. 4A), recapitulating the association between WGD clonal fraction and CIN scores observed in single cells (Fig. 1, D and G).

**Fig. 4.**
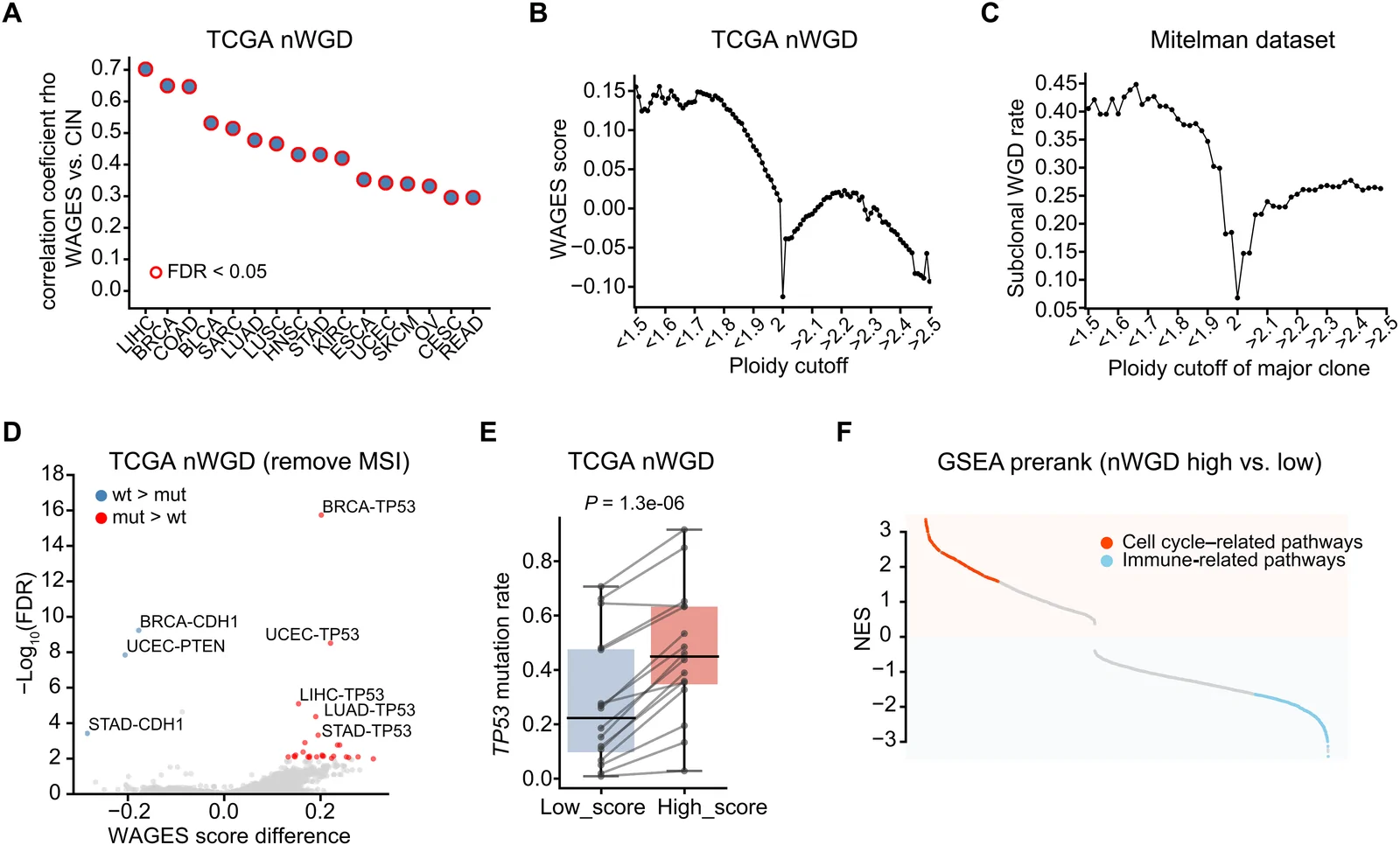
Genomic correlates of WAGES score in nWGD tumors. (**A**) Correlation between the WAGES score and CIN scores in TCGA nWGD tumors. Each dot represents one cancer type. ρ and *P* values are calculated using Spearman correlation. All the correlations are significant (FDR of <0.05). (**B**) Distribution of the WAGES score across tumor ploidy in TCGA nWGD tumors. For hypodiploid nWGD tumors (ploidy of <2), each dot represents the average WAGES score of tumors with ploidy lower than the corresponding value on the *x* axis. For hyperdiploid nWGD tumors (ploidy of >2), each dot represents the average WAGES score of tumors with ploidy higher than the corresponding value. The dot at ploidy 2 shows the average score of tumors with ploidy between 1.99 and 2.01. (**C**) Frequency of WGD subclones across solid tumor ploidy based on multiclone karyotyping data from the Mitelman database. Each dot represents the frequency of nWGD tumors harboring a WGD subclone, computed cumulatively across ploidy as in (B). (**D**) Association between WAGES score and gene mutations in TCGA nWGD tumors. *x* axis, score difference between mutant and wild-type samples. *y* axis, FDR at log_10_ scale. *P* values are calculated using two-sided Wilcoxon rank sum test and are adjusted by Benjamini-Hochberg. Genes with FDR of <0.01 and score differences of >0.1 are considered significant. Red, score higher in mutants (mut); blue, score higher in wild type (wt). (**E**) *TP53* mutation rate in high-score and low-score nWGD tumors. Each dot represents a cancer type. *P* values are calculated using paired *t* test. (**F**) Pathway analysis between high and low score nWGD tumors. Each dot represents a pathway. *y* axis, normalized enrichment score (NES). Red, significant cell cycle–related pathways (FDR of <0.05); blue, significant immune-related pathways (FDR of <0.05).

We next examined WAGES scores across tumor ploidy (Materials and Methods). WAGES scores increased in tumors deviating from diploidy (Fig. 4B), and the increase was particularly marked in low-ploidy tumors, implying a role of deletion in fostering WGD. To validate this pattern, we used multiclonal cytogenetic data from the Mitelman database (Materials and Methods) (*32*). From nWGD tumors where at least two clones with distinct karyotypes were observed, we counted the percentage of tumors harboring a WGD subclone (Fig. 4C). The pattern from the Mitelman dataset resembled the pattern calculated from the WAGES score (Fig. 4, B and C). This resemblance provides further support for WAGES as a measurement of WGD cell presence in nWGD tumors.

Given WAGES’s ability to detect WGD presence, we sought to identify its genomic correlates for more evidence of WGD subclones in patient nWGD tumors. We first correlated the WAGES score with gene mutations. For each gene, we compared WAGES scores between mutant and wild-type tumors. In total, we identified 415 negative correlations (FDR of <0.01, score difference of <−0.1) and 27 positive correlations (FDR of <0.01, score difference of >0.1; fig. S6A). However, 94% of the correlations, mainly negative correlations, were attributable to tumors with microsatellite instability (MSI), consistent with their mutual exclusivity with WGD (*6*, *15*). After excluding MSI tumors, 29 significant correlations remained (positive, *n* = 26; negative, *n* = 3) (Fig. 4D and table S3).

The 29 correlations involved 23 genes, which can be functionally categorized into p53, mitotic spindle, and cell-cell adhesion pathways. Evidence from the literature linked at least 14 of the 23 genes to cell cycle regulation or WGD (table S3). The most frequently identified gene was *TP53*, mutations of which were associated with higher WAGES scores in six cancer types. In a separate analysis, we divided nWGD tumors into high and low-score groups and observed elevated *TP53* mutation rates in high-score tumors in 15 of the 16 cancer types tested (*P* = 1.3 × 10^−6^, paired *t* test; Fig. 4E). These results were consistent with *TP53* mutations providing permissive context for WGD cells. *CDH1* mutations were associated with low WAGES scores in breast and stomach cancers (Fig. 4D), and the association can be explained by disease histology (fig. S6B).

Mutations of several long genes including *TTN* and *MUC16* were also associated with higher WAGES scores. These genes are prone to mutations due to the length and late replication timing (*33*) and likely accumulate more mutations in evolutionarily advanced tumors. Previous studies reported a tight correlation between their mutation rates and overall tumor mutational burden (TMB) (*34*, *35*). Consistently, we observed higher TMB in high-score nWGD tumors with borderline significance (*P* = 0.11, TCGA; *P* = 0.07, TRACERx lung; fig. S6, C and D).

We next compared gene expression between high- and low-score nWGD tumors. Pathways up-regulated in the high-score group were predominantly involved in cell cycle. Pathways down-regulated were enriched for immunity-related functions (Fig. 4F and table S4). These transcriptomic differences mirrored previously reported differences between WGD and nWGD tumors (*36*).

### WAGES score correlates with metastatic potential, treatment responses, and prognosis

To gather insights into the clinical significance of subclonal WGD cells, we associated the WAGES score with tumor metastatic potential, treatment responses, and prognosis. In lung adenocarcinoma (LUAD), the prevalence of WGD is higher in metastatic diseases (*13*); thus, a subset of metastatic WGD tumors may trace their origin to nWGD primary tumors. Using TRACERx lung data (*37*), we found that, among primary nWGD tumors, WAGES scores were significantly higher in patients that developed metastatic tumors during follow-up (>3 years) than patients who did not (*P* = 1.8 × 10^−4^, Wilcoxon rank sum test; Fig. 5A), suggesting an association between WAGES scores and metastatic potential. As control, we also analyzed primary nWGD lung squamous cell carcinoma (LUSC). Different from LUAD, the prevalence of WGD is similar between primary and metastatic diseases in LUSC (*13*). No significant association was observed between WAGES scores and metastatic potential for LUSC (*P* = 0.35, Wilcoxon rank sum test; Fig. 5A).

**Fig. 5.**
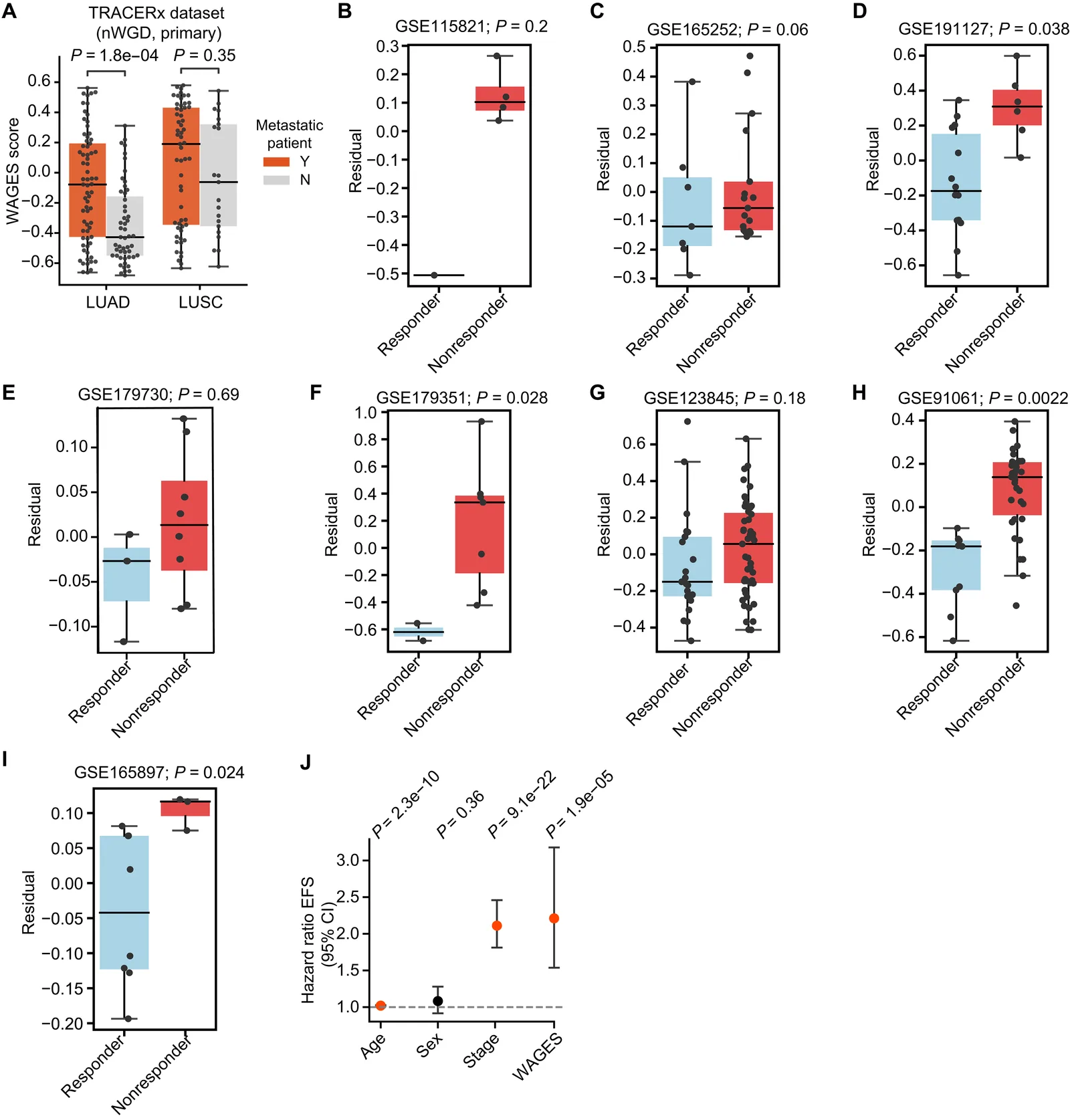
Clinical significance of the WAGES score. (**A**) WAGES score associates with metastatic potential in lung adenocarcinoma (LUAD), but not lung squamous cell carcinoma (LUSC). Each dot represents a primary nWGD tumor. Nonmetastatic patients are defined as those who did not develop metastasis, recurrence, progression, or any new primary tumors in at least 3 years of follow-up. *P* values are calculated using two-sided Wilcoxon rank sum test. The boxes and middle lines within represent the IQR and median. The top and bottom whiskers represent values within 1.5× IQR of the upper and lower quartiles, respectively. Box plots in other panels are interpreted similarly. (**B** to **I**) Correlation of WAGES score residual with patient response to treatments. Each plot represents a longitudinal dataset. Responders and nonresponders are derived from the original study or defined based on clinical data if not defined in the original study. *P* values are calculated using one-sided Wilcoxon rank sum test. (**J**) Association between WAGES score and prognosis in TCGA nWGD tumors after adjusting for tumor stage, patient age, sex, and cancer type. *P* values are calculated using multivariable Cox proportional hazards regression. Red dots indicate statistical significance (*P* < 0.05). Whiskers denote 95% confidence interval (CI).

We next associated WAGES scores with tumor responses to treatments. The mainstay of cancer therapy, including chemotherapy and increasing immunotherapy, exerts potent selection pressure on the heterogeneous cancer cell population. The selection can lead to changes in tumor clonal composition over the course of treatment. Because the WAGES score can reflect WGD clonal fraction, we analyzed pre- to posttreatment changes in WAGES scores and evaluated how these changes relate to therapeutic response.

We assembled eight longitudinal datasets with paired pre- and post- or on-treatment samples for each patient (table S5). Patients across these datasets received a range of therapies, including neoadjuvant chemotherapy and immune checkpoint blockade therapy. For each patient, we calculated a WAGES residual, which captures changes in WAGES scores after adjusting for the baseline pretreatment value (Materials and Methods). Direct correlations with raw WAGES scores could conflate baseline heterogeneity with therapy-induced changes; using the residual isolates the treatment-associated component of the signature that is more informative of tumor response.

Across all eight datasets, WAGES residuals were higher in nonresponders than in responders (Fig. 5, B to I). Although some datasets had small sample sizes, the consistent direction of effect across cohorts strongly supports an association between WAGES scores and tumor response. Accordingly, meta-analyses using both Stouffer’s and Fisher’s methods yielded significant results (*P* = 3.2 × 10^−5^ and *P* = 8.8 × 10^−5^, respectively).

Last, we associated WAGES with patient prognosis. WGD was previously shown to confer worse patient outcomes compared with nWGD tumors (*7*, *15*). However, in 8 of the 13 cancer types that we tested, patients with high-score nWGD tumors demonstrated worse or comparable event-free survival (EFS) than patients with WGD tumors (fig. S7). In individual cancer types, multivariable analysis controlling for patient age, sex, and disease stage confirmed the association between the signature and EFS in nWGD tumors (fig. S8A). Across pan-cancer, controlling for cancer type and the same set of confounders confirmed the independent prognostic value of the signature in nWGD tumors (*P* = 1.9 × 10^−5^, HR = 2.2, 95% confidence interval of 1.5 to 3.2, Cox proportional hazards model; Fig. 5J). Adding additional covariates including tumor purity, proliferation, CIN, and *TP53* mutation status to the model either alone or in combination retained the significance, with attenuation in the fully adjusted model (table S6). The association between the WAGES score and patient EFS was much weaker in WGD tumors (*P* = 0.07, Cox proportional hazards model; fig. S8B), reiterating the relevance of the signature to nWGD, but not WGD, tumors. This observation was replicated using the TRACERx lung cancer dataset (fig. S8, C and D).

## DISCUSSION

In this study, we provide evidence that WGD cells are present not only in WGD tumors but also in tumors previously classified as nWGD. While the presence of WGD cells is expected in WGD tumors, their detection in nWGD tumors is notable, as the fraction of WGD cells observed far exceeds what would be expected if WGD was a neutral genetic event (*12*). In WGD cells, the positive correlation between WGD clonal fraction and SCNA burden suggests that the WGD subclones are subject to ongoing chromosomal instability. This correlation is primarily driven by postWGD SCNAs, consistent with the notion that genome doubling provides a permissive environment for the accumulation of additional chromosomal alterations (*6*, *7*, *15*).

To extend our single-cell findings to bulk tumors, we developed a gene signature called WAGES. We validated WAGES using multiple independent datasets spanning technological platforms and cancer types. In scDNA-seq data, WGD cells show largely coherent SCNA patterns, suggesting that cells of the same pattern may share the same cellular origin; thus, we posit that WAGES reflects WGD clones rather than sporadic WGD cells. Analyses of single-cell and spatial transcriptomic data showed that WAGES not only is specific to cancer cells but also reflects WGD clonal fraction. This characteristic makes WAGES a useful tool to stratify bulk nWGD tumors into subgroups that harbor different proportions of WGD subclones. The same characteristics, however, prompt caution for using WAGES as a prediction tool to distinguish WGD and nWGD tumors, because tumor purity and presence of WGD subclones in nWGD tumors can affect WAGES scores. Note that WAGES was mostly tested in cancer datasets. Further validation in the noncancer context is warranted to better define its applicability.

Applying WAGES to bulk nWGD tumors, we observed significant correlations between signature scores and SCNA burden, recapitulating the associations seen at the single-cell level. Further, unsupervised analysis of WAGES in nWGD tumors revealed associations with genomic features including *TP53* mutations, high proliferative activity, and reduced immune signaling that parallel the differences observed between WGD and nWGD tumors (*36*). Together, these findings substantiate the single-cell observation that WGD subclones are likely common in nWGD tumors.

Clinically, the presence of WGD cells in nWGD tumors is concerning because the heterogeneity can facilitate clonal selection under potent selective pressures such as cancer therapies. Previous studies have shown that WGD can suppress immune activation (*12*, *38*) and confer resistance to chemotherapy and various targeted therapies (*16*, *17*, *39*, *40*). Consequently, treatments that eliminate nWGD cells may inadvertently select for WGD subclones. Consistent with this hypothesis, higher WAGES scores were associated with poor responses to cancer therapies in longitudinal datasets, as well as worse patient outcomes. We note that ongoing chromosomal instability introduces substantial heterogeneity within WGD populations, and, thus, responses of WGD cells to therapy are likely heterogeneous.

Despite the extensive validation, WAGES described in this study can only indirectly estimate the prevalence of WGD cells in nWGD tumors. Definitive evidence will need to be collected from more scDNA-seq experiments, ideally across more cancer types and disease stages including pre-cancer. Nonetheless, we present evidence for the common presence of active WGD subclones in nWGD tumors.

## MATERIALS AND METHODS

### scDNA-seq and scRNA-seq data processing

We obtained scDNA-seq and scRNA-seq data from https://zenodo.org/records/6998936 (*25*). This dataset consists of >55,000 single cells from 34 samples, including 16 high-grade serous ovarian cancer patient-derived xenografts (PDXs), 9 triple-negative breast cancer PDXs, 1 ovarian cancer cell line, and 8 normal 184-hTERT cell lines. The normal cell lines were not used in our analysis. These data were high quality cells generated by DLP^+^ platform (*41*), which performs strict quality control to exclude doublets and S-phase cells.

We identified WGD cells in the scDNA-seq dataset using a method adapted from previous studies (*12*, *15*). Briefly, we first identified chromosome arms with at least 70% segments showing major allele copy number (major_CN) of ≥2. We classified a cell as WGD if half or more of the chromosome arms met the criterion. In total, we identified 27,484 WGD cells and 12,550 nWGD cells. For simplicity, we excluded 4081 WGD cells with ploidy of >4 from the following analyses, because these cells likely underwent two WGD events. For a given chromosome (1−22 and X), the chromosome specific ploidy was estimated as

$$\text{Ploidy}=\frac{\sum_{k=1}^{n}l_{k}\times {\text{CN}}_{k}}{L}$$

where L is the total length of all segments, $l_{k}$ is the length of a given segment k, *n* is the number of segments, and ${\text{CN}}_{k}\;$ is the absolute copy number of the corresponding segment. Cell ploidy was calculated as the average ploidy across all chromosomes.

Chromosomal instability (CIN) score was calculated as the average fraction of chromosome arms with copy number alterations, similar to fraction of the genome altered. It captures the effect of focal and broad events on altering the genome without the need to balance the altitudes of gains and losses. For the calculation of a tumor’s overall CIN score, copy number alterations were defined as deviations from the baseline absolute copy number level (2 for nWGD and 4 for WGD). For WGD cells, we further timed copy number segments into preWGD and postWGD using parsimonious rules published previously (*15*). The principle of parsimony assumes that cells arrive at a certain copy number state using the shortest route. For instance, to reach genotype AAB in a WGD tumor, possible routes can be “AB -> AABB -> AAB” or “AB -> AABB -> AAABB -> AAAB -> AAB.” The parsimonious rules would select the first route because it entails fewer steps. Copy number profiles from the timing analysis were used for preWGD and postWGD CIN score calculation. To illustrate this calculation, we continue to use the above toy example. The overall CIN score of the genotype AAB is 1 because, compared with the “normal” genotype AABB, the segment lost one copy of B. In the first route, the preWGD CIN is 0 as no alteration occurs preWGD. The postWGD CIN is 1 for this segment as deletion of the B allele occurs postWGD. SCNA similarity between WGD and nWGD cells was calculated using Pearson correlation based on the averaged absolute copy number profiles of the nWGD or WGD cells from each patient.

### Mapping the WGD status of scDNA-seq to scRNA-seq data

In the Funnell dataset, 19 of the 26 cancer samples with scDNA-seq data also have matched scRNA-seq data. To identify WGD cells from scRNA-seq, we inferred relative copy number profiles using scRNA-seq and compared with those from scDNA-seq. For scRNA-seq data, we applied copyKAT (*42*) and SCEVAN (*43*) to infer relative copy number profiles and corresponding copy number clusters. We used the following rules to map WGD status in scRNA-seq:

1) For WGD cells with specific copy number changes at the scDNA level, if we also observed the same copy number changes in one of the clusters identified by SCEVAN or in the aneuploidy group identified by copyKAT, then we took all cells of this group as WGD cells in scRNA data.

2) For those without specific copy number changes, if the normal cluster identified by SCEVAN matched with the copyKAT diploid cluster, then we treated this group as nWGD cells in scRNA data.

3) For samples that cannot be mapped by approaches mentioned above, we obtained haplotype specific CNAs identified by Funnell *et al.* (*25*). We then mapped the WGD-specific changes of scDNA-seq to scRNA-seq based on haplotype specific changes. Here, we limited this analysis to six samples with sufficient WGD and nWGD cells in scDNA-seq (WGD cell fraction 10 to 90%). We confidently mapped cell WGD status for three samples (OV2295, SA1184, and SA1052).

### Identification of WAGES

To identify genes associated with WGD, we downloaded gene expression matrices and WGD status information from TCGA PanCanAtlas (https://gdc.cancer.gov/about-data/publications/pancanatlas) and PCAWG (http://dcc.icgc.org/pcawg/). To control for variation caused by unknown factors in the data, we performed principal components analysis (PCA) on gene expression data. We examined pairwise correlation among the top 20 components and did not observe noticeable collinearity. A linear regression model was applied to TCGA and PCAWG datasets with the formula “gene expression ∼ WGD status + tumor purity + cancer type + cancer stage + PCs.” For the PCAWG dataset, cancer stage was not available. For the TCGA dataset, we used samples that have consensus ploidy values predicted by ABSOLUTE (*44*) and ASCAT (*45*) (difference of <0.5). We also excluded tumors with two doublings. The ABSOLUTE and ASCAT copy number data were downloaded from PanCanAtlas and COSMIC (version 87), respectively.

Genes with WGD variable coefficient of >0 and FDR of <0.05 were identified as WGD associated genes. To increase confidence, we intersected the genes identified from TCGA and PCAWG datasets. This led to a set of 598 WGD associated genes. Based on this gene set, we calculated signature scores for bulk RNA sequencing (RNA-seq) using gene set variation analysis (GSVA) (*46*). For scRNA-seq, score_genes function of scanpy package (*47*) was used to calculate the signature score. A gene signature specific to nWGD tumors was similarly generated for quality control purposes (see below).

### Validation of WAGES

For the TCGA dataset, we included cancer types with at least 20 nWGD and 20 WGD samples, and cancer types with WGD rate between 10 and 90%. We excluded glioblastoma from the analysis because the WAGES score did not demonstrate the anticipated anticorrelation with the nWGD signature score. These filtering steps resulted in 17 cancer types. Among them, lower grade glioma (LGG) did not show higher WAGES scores in WGD tumors in the verification. We thus excluded LGG in downstream analyses due to concerns over the signature’s validity in this cancer type.

Validation datasets were downloaded from CCLE, Genomic Data Commons (GDC; version 43.0), and Gene Expression Omnibus (GEO). For GDC datasets, we limited to solid tumor datasets with absolute copy number segmentation profiles. Then, only three datasets (CGCI-HTMCP-CC, CGCI-HTMCP-LC, and CPTAC-3) with at least 10 nWGD and 10 WGD samples were kept for analysis. WGD status was determined on the basis of the fraction of the genome with major allele copy number of ≥2 (*15*).

We collected other bulk RNA-seq datasets with ploidy information, including diploid and isogenic tetraploid RPE1 cells (GSE86101 and GSE86102) (*27*) and colorectal cell line DLD-1 (GSE81394) (*28*). The scRNA-seq validation dataset (GSE222389) included parental diploid and isogenic tetraploid *TP53*^−/−^ RPE1 cells sampled at 6 and 20 weeks post–WGD induction (*10*).

### WAGES and WGD clonal fraction in simulated datasets

To test whether WAGES can measure WGD clonal fraction, we simulated bulk samples with known WGD clonal fractions. We leveraged the samples from which we have confidently determined cell WGD status in the scRNA-seq data. At a WGD fraction, we randomly sampled and mixed a total of 1000 WGD and nWGD cells from each sample to form a pseudobulk. The WGD fraction ranged from 0 to 1 with a step of 0.1. For each fraction, we generated 20 replicates. Pseudobulk gene expression was generated by summing up the expression of all cells for each replicate. DESeq2 (*48*) was applied to normalize the expression data. We calculated WAGES by GSVA based on the normalized pseudobulk gene expression matrix. Sample SA1184 was not used in this analysis because the number of cells was too small for the random sampling.

### WAGES in spatial transcriptome data

The curated single-cell spatial transcriptome dataset was obtained from a published study (*30*). This dataset comprises 137 spatial transcriptomic slides from different cancer types, and the study has quantified expression activity for more than 40 cell types. Colocalization of WAGES and other cell signatures was done using spotwise Pearson correlation across samples.

To further validate the colocalization between WAGES and cancer cells, we downloaded an independent spatial transcriptomics dataset (*49*). The dataset also has pathological annotation. We calculated WAGES scores and compared them with pathology annotation.

### Pan-cancer analysis of WAGES in nWGD tumors

To identify gene mutations associated with WAGES, we compared WAGES scores between mutant and wild-type samples for each gene using two-sided Wilcoxon rank sum test. We used Benjamini-Hochberg method for multiple hypothesis testing. Genes with a minimum score difference of 0.1 and FDR of <0.01 were considered significantly associated with WAGES. We limited to genes with mutations in at least five samples and a minimum mutation rate of 5% in each cancer type.

To associate WAGES with other genomic features, we correlated WAGES scores with tumor ploidy, CIN, aneuploid score, and TMB. The aneuploid score, TMB, and MSI/MSS information were downloaded from previous studies (*50*–*52*).

To correlate gene expression with the signature, we divided nWGD tumors into high-score and low-score groups by the median value of the signature scores in each cancer type. We ranked the genes based on Cohen’s and applied the preranked GSEA to obtain pathways enriched in high- and low-score samples.

### Clinical data analyses

To associate WAGES with cancer treatment response, we collected eight longitudinal datasets (*53*–*60*). We only used paired samples collected pre-, on-, or posttreatment, mostly neoadjuvant chemotherapy or immunotherapy, from 191 patients. WAGES scores were calculated from either bulk sequencing data (GSE115821, GSE165252, GSE191127, GSE179730, GSE179351, GSE123845, and GSE91061), or pseudobulks constructed from scRNA-seq data (GSE165897).

For each study, we calculated differences in WAGES score between pre- and post/on-treatment across patients. To adjust for patient baseline WAGES score, we calculated WAGES residual as the difference between the observed post/on-treatment WAGES score and the fitted post/on-treatment WAGES score with pretreatment as the independent variable using general linear-regression model. The residual reflects treatment-associated WAGES changes because it removes the baseline differences. The linear regression residual was calculated using python package scikit-learn (version 1.3.2) (*61*).

Patient categorization into responders and nonresponders were derived from the source for most studies. For dataset GSE191127, responders and nonresponders were classified on the basis of patient recurrence status because no response labels were provided in the study. For datasets GSE179351 and GSE91061, patient response was rated using the RECIST criteria. We classified responders as patients who showed partial response or complete response, nonresponders as patients who showed progressive disease or stable disease.

We further tested the prognostic value of WAGES in the TCGA datasets. Differences in EFS were compared between high-score and low-score nWGD tumors using the Kaplan-Meier method, and *P* values were calculated using log-rank test. To adjust for other confounding factors, we applied multivariable survival analysis using Cox proportional hazards models (survival ∼ WAGES + stage + age + sex). In pan-cancer analysis, “cancer type” was added as an additional variable. Other variables, including CIN scores, tumor purity, *TP53* mutation status and proliferation (*62*), were also tested. Tumor stage here was categorized as early stage (I/II) and late stage (III/IV) to increase statistical power. We excluded SARC, READ, and ESCA from the Cox regression analysis because of small sample sizes or lack of stage information. Survival analyses for TRACERx lung data were similarly done except adding smoking status as a confounding factor.

### Other analyses

TCGA copy number profile, expression matrix, and somatic mutation data were downloaded from PanCanAtlas (https://gdc.cancer.gov/about-data/publications/pancanatlas). All statistical analysis was performed using SciPy (version 1.9.3) or statsmodels package (version 0.14.0) in python (*63*, *64*). Expression normalization was conducted by PyDESeq2 (version 0.4.4) (*65*). Survival analysis and Cox regression were performed using lifelines python package (version 0.27.4) (*66*). The python package GSEApy (version 1.1.1) (*67*) was used for enrichr, GSEA, and GSVA analysis. The gene set list used for gene enrichment was downloaded from MSigDB dataset (c2.cp.v2023.2.Hs.symbols.gmt). All figures were generated by python package matplotlib (version 3.5.2) and seaborn (version 0.11.2) (*68*, *69*).
