# Supplementary material for: Evidence of active whole-genome doubling (WGD) subclones in non-WGD tumors
Source: Sci Adv. 2026 Oct 30;12(44):eaee7939. doi: 10.1126/sciadv.aee7939 (PMC13722003; doi:10.1126/sciadv.aee7939)
Supplement: Supplementary file 1 [file sciadv.aee7939_sm.pdf]

Science Advances
AAAS

Supplementary Materials for

Evidence of active whole-genome doubling (WGD) subclones in non-WGD tumors

Funan He *et al.*

Corresponding author: Siyuan Zheng, zhengs3@uthscsa.edu

Sci. Adv. **12**, eaee7939 (2026)
DOI: 10.1126/sciadv.aee7939

The PDF file includes:

Figs. S1 to S8
Legends for tables S1 to S6

Other Supplementary Material for this manuscript includes the following:

Tables S1 to S6

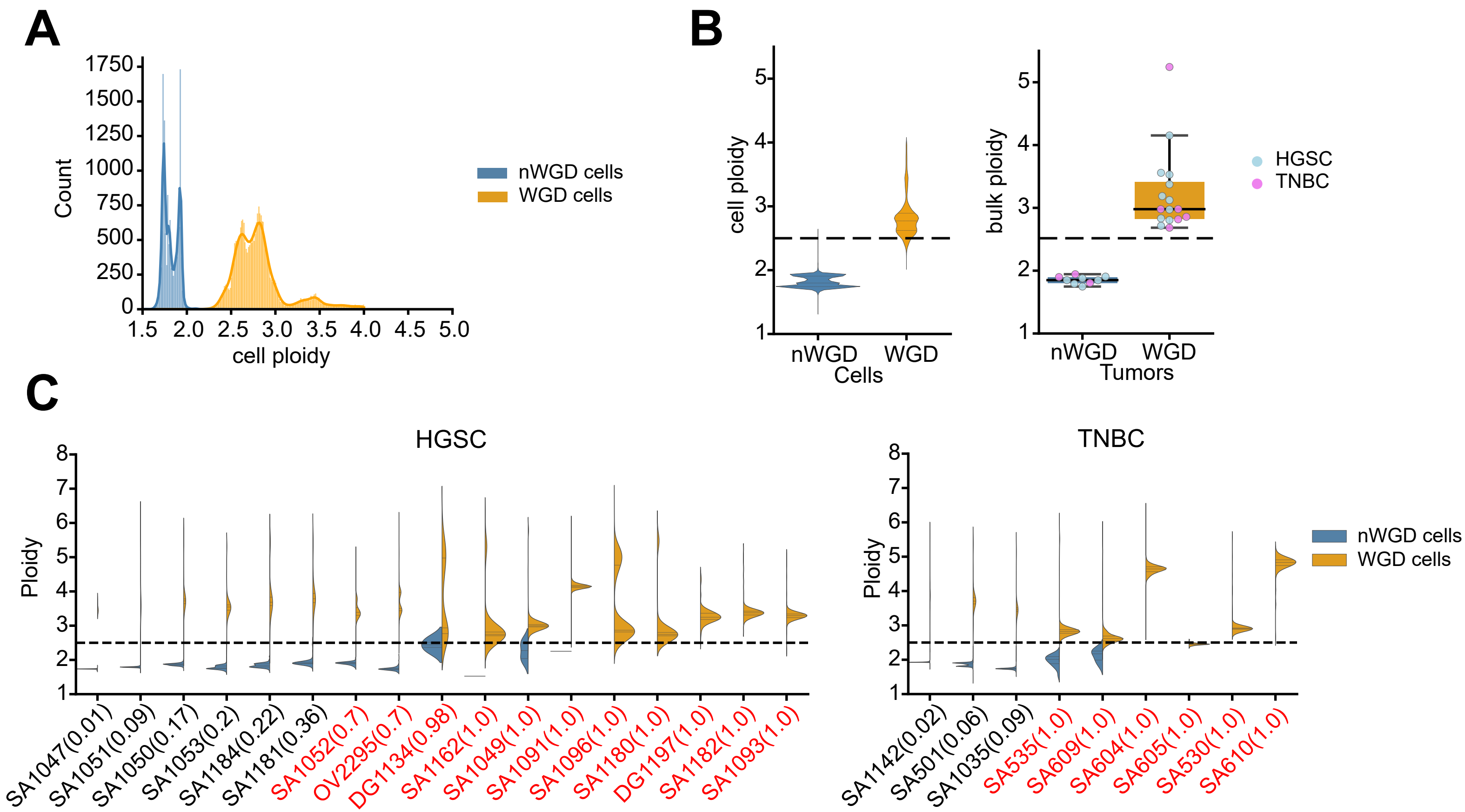

Fig. S1. Ploidy of single cells. A, Distribution of cell ploidy. **B**, Ploidy of WGD and nWGD cells and the matched bulk tumors. The bulk ploidy is obtained from the bulk tumor WGS. Each dot represents one tumor in the bulk sample data. HGSC, high-grade serous ovarian cancer. TNBC, triple-negative breast cancer. The boxes and middle lines within represent the interquartile range (IQR) and median. The top and bottom whiskers represent values within 1.5×IQR of the upper and lower quantiles respectively. **C**, Cell ploidy stratified by sample. The numbers in parentheses show WGD clonal fraction, i.e., fraction of WGD cells in the sample. Samples highlighted in red are WGD tumors. Note that in samples DG1134, SA1049, SA535, SA609 there appears to be a large blue shade indicating many nWGD cells. This is a violin plot artifact. Very few nWGD cells were detected in these tumors.

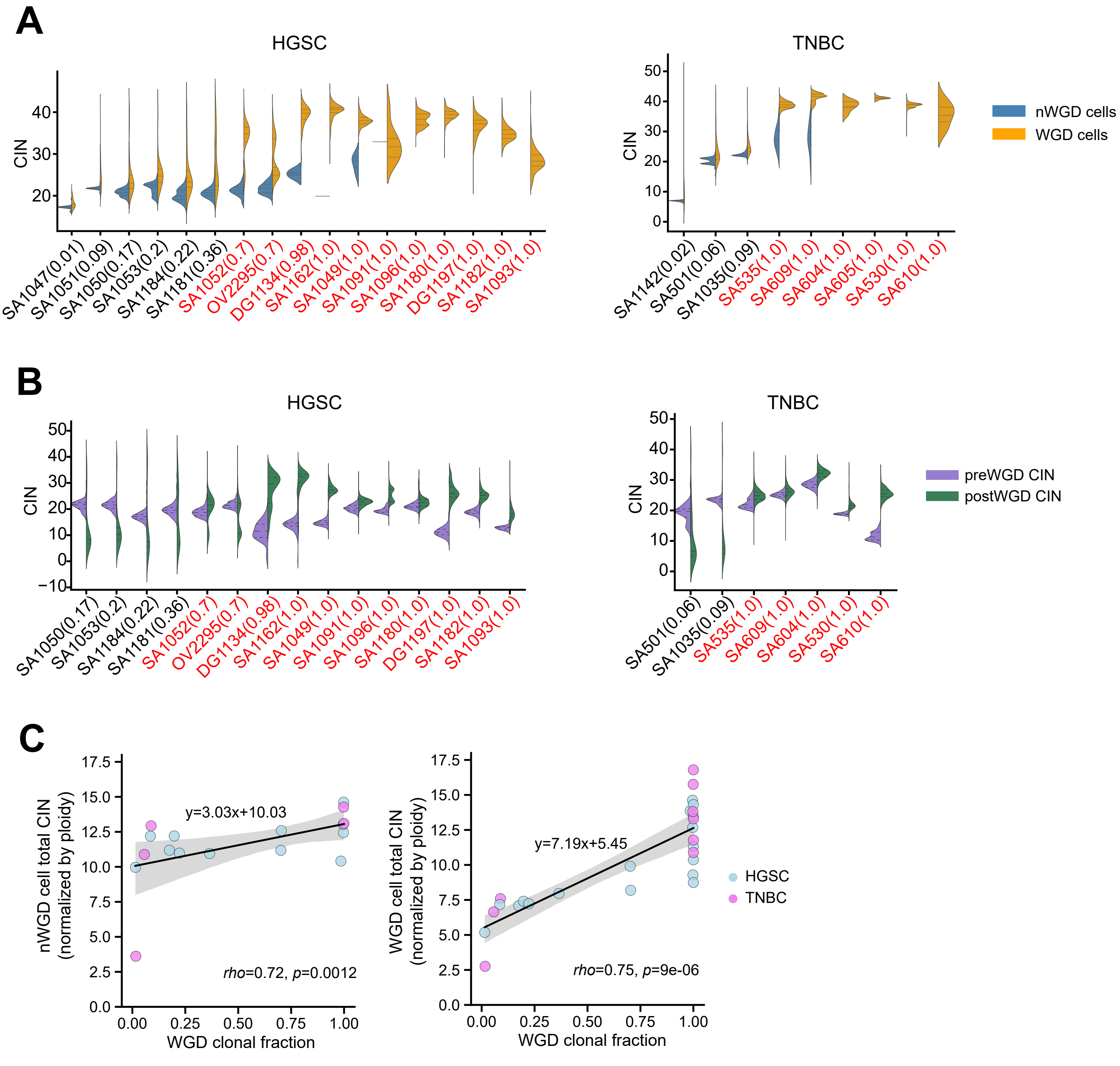

Fig. S2. Chromosomal instability (CIN) score of single cells. A, Distribution of cell total CIN scores across samples. Numbers in parentheses represent WGD clonal fraction. Samples highlighted in red are WGD tumors. **B**, Distribution of pre and postWGD CIN scores across samples. **C**, Correlation between WGD clonal fraction and ploidy-normalized CIN scores of nWGD (left) and WGD cells (right). Rho and p values are calculated using Spearman correlation, and slope and intercept are calculated using linear regression. Each dot represents one sample. Shade represents 95% confidence interval.

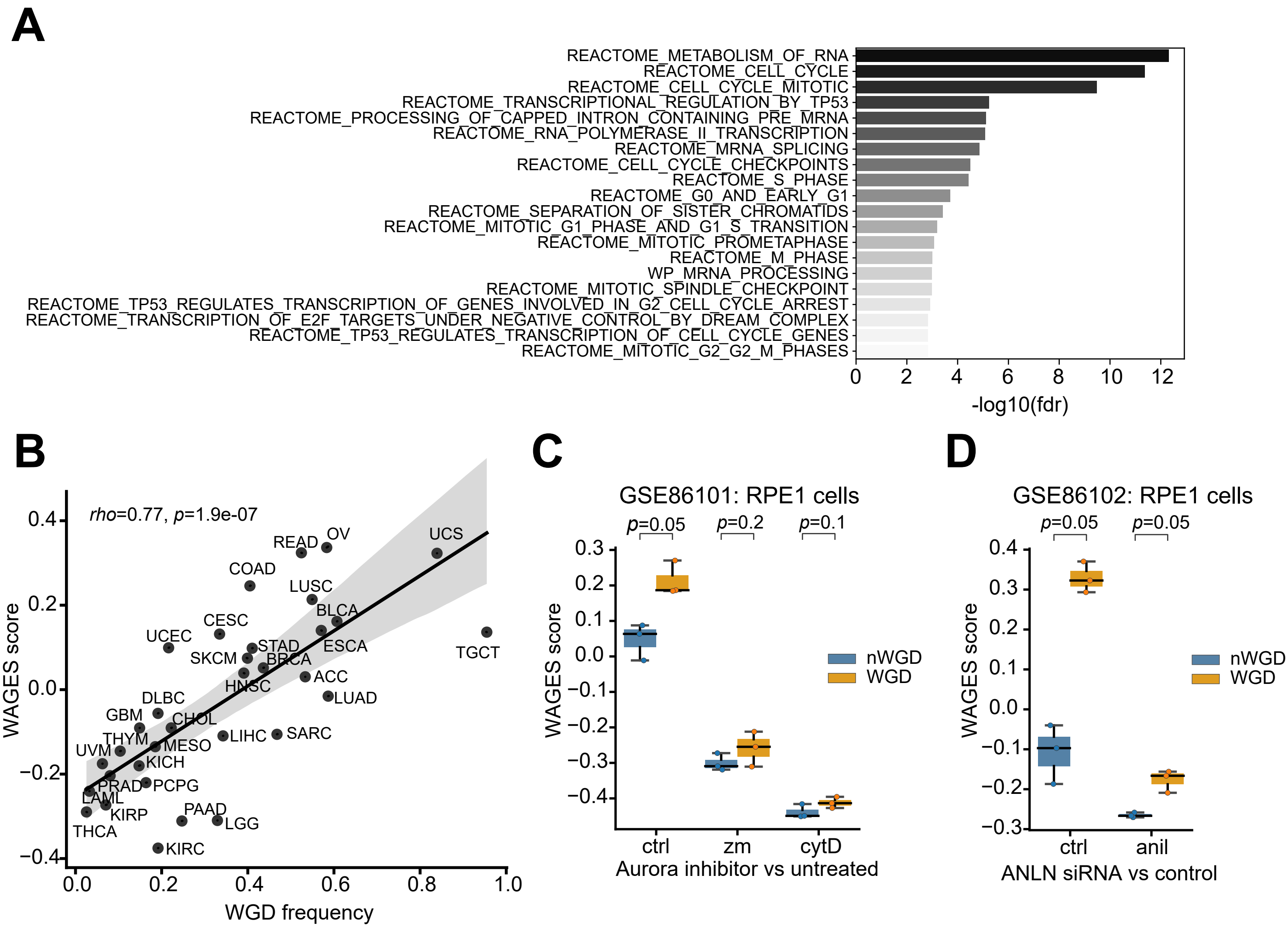

Fig. S3. Characterization and validation of WAGES. **A**, Top 20 pathways enriched with the WAGES genes. **B**, Correlation between WAGES scores and WGD frequencies on cancer type level. Rho and p values are calculated using Spearman correlation. Cancer types with at least 20 samples were included, and each dot represents a cancer type. **C-D**, WAGES score stratified by cell ploidy status and WGD induction methods in GSE86101 (C) and GSE86102 (D) Zm, ZM447439, an aurora kinase inhibitor; cytD, cytochalasin D, an inhibitor of actin polymerization; anil, anillin knockdown with siRNA. P values are calculated using one-sided Wilcox rank sum test.

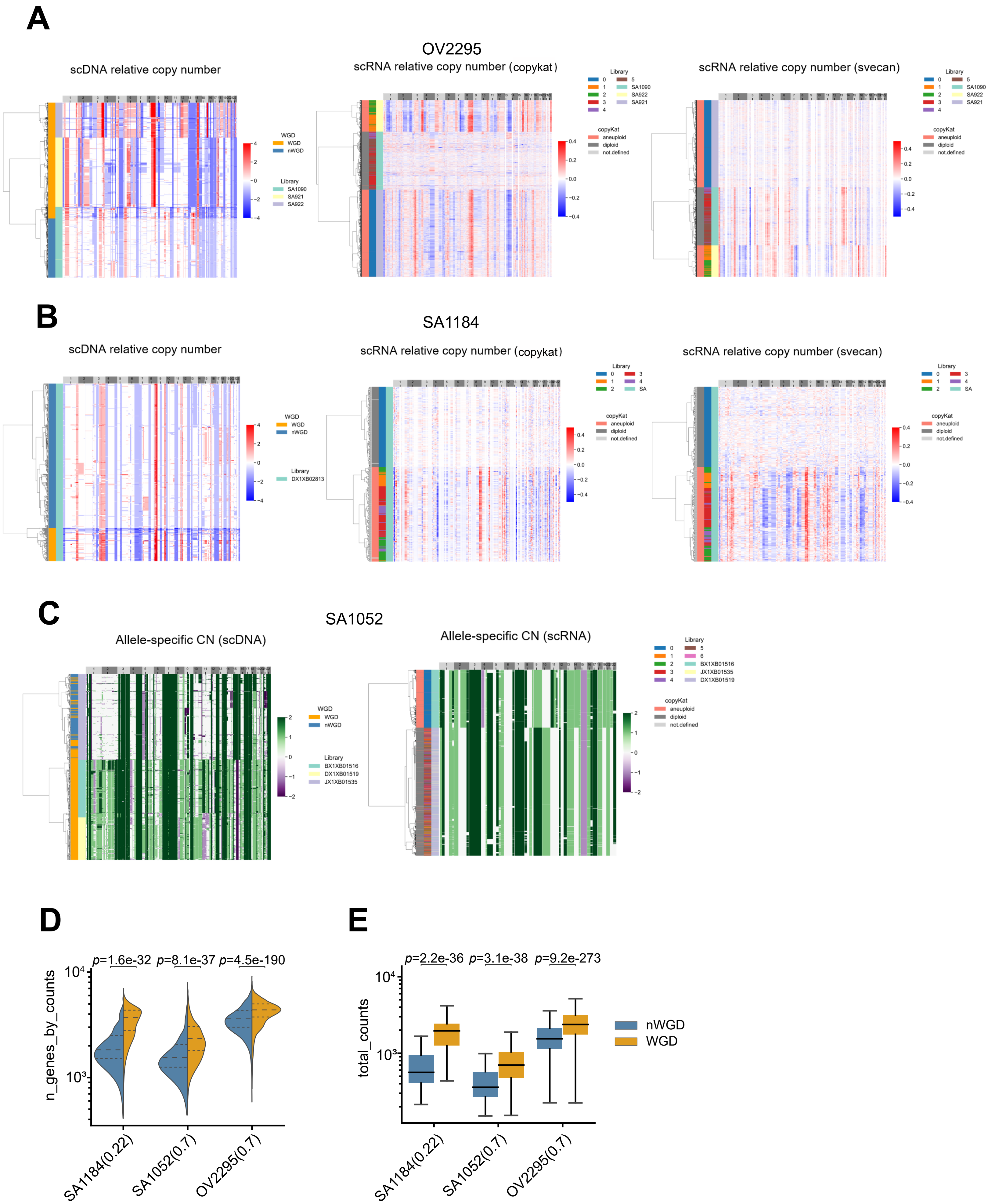

Fig. S4. Mapping WGD cells from scDNAseq to scRNAseq data. **A**, OV2295. Left, relative copy number profile from scDNAseq. Middle, relative copy number profile from scRNAseq inferred with copykat. Right, relative copy number profile from scRNAseq inferred with SCEVAN. **B**, SA1184, similar to A. **C**, SA1052. Left and right panels show allele specific copy number pattern estimated with SIGNAL. **D**, Number of gene detected in scRNAseq. Numbers in parentheses on x axis denote WGD clonal fraction. **E**, Total read counts in presumably WGD and nWGD cells. P values are calculated using two-sided Wilcox test. Boxplots are interpreted similarly as in other figures.

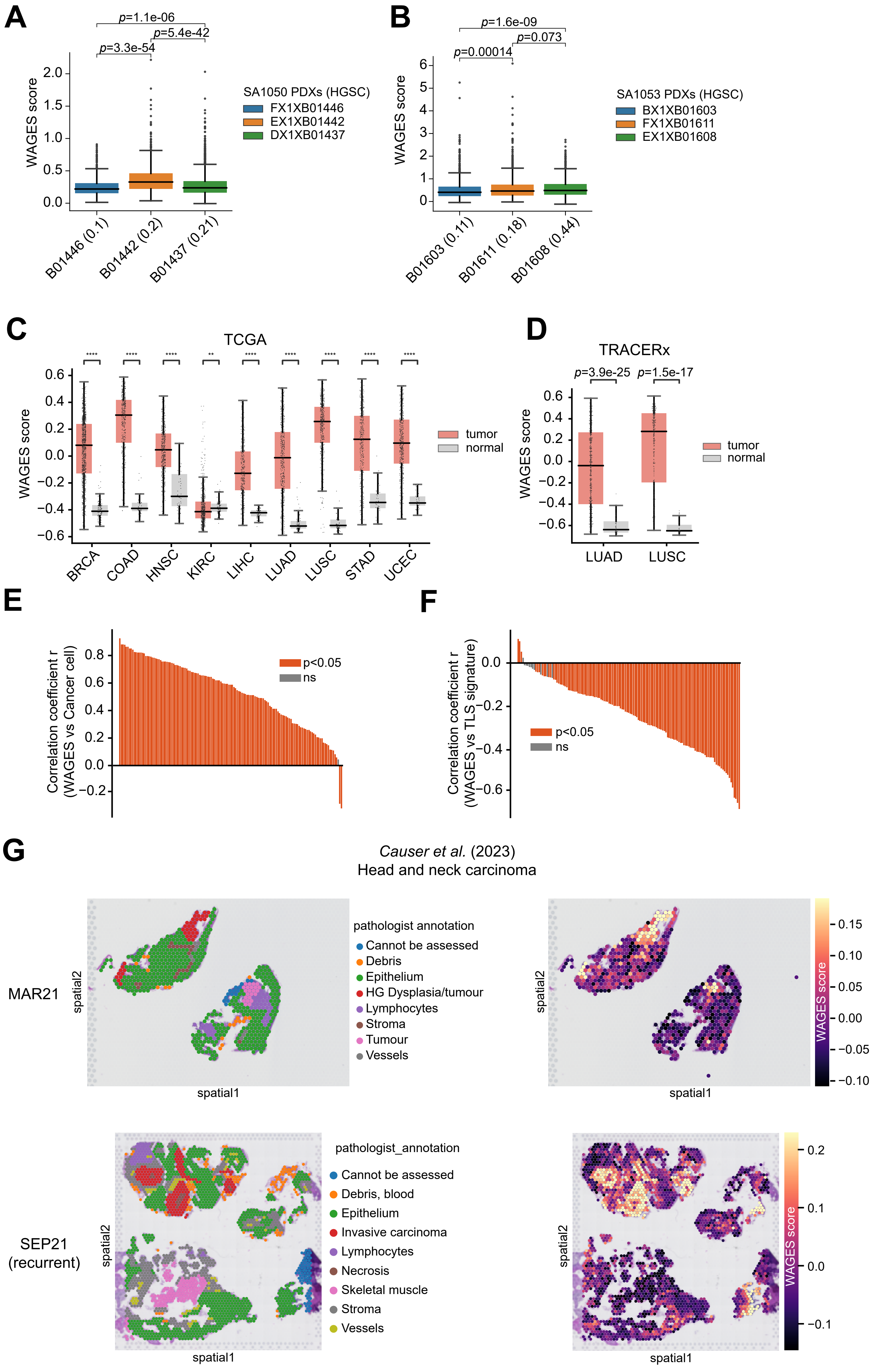

Fig. S5. WGD signature, cancer cell specificity, and WGD clonal fraction. A-B, WAGES score across isogenic samples with varying WGD clonal fractions. Numbers in parentheses show WGD clonal fraction. P values are calculated using two-sided Wilcox test. Boxplots are interpreted similarly as in other figures. **C-D,** WAGES score in tumor and normal samples from TCGA (C) and TRACERx lung (D). Nine cancer types with at least 20 tumor and 20 normal samples in TCGA were included. P values are calculated using two-sided Wilcox test (*, p≤0.05; **, p≤0.01; ***, p≤0.001; ****, p≤0.0001). Each dot represents one sample. **E,** Pearson correlation coefficient between the WAGES score and cancer cell signature across 137 spatial transcriptome slides. Each bar represents one slide. **F,** Pearson correlation coefficient between the WAGES score and tertiary lymphoid structure (TLS) signature across 137 spatial transcriptome slides. **G**, Co-localization of the WAGES score and cancer cells in a pathologically annotated head and neck carcinoma patient. The left panel shows pathological annotation of cell types. The right panel shows WAGES score.

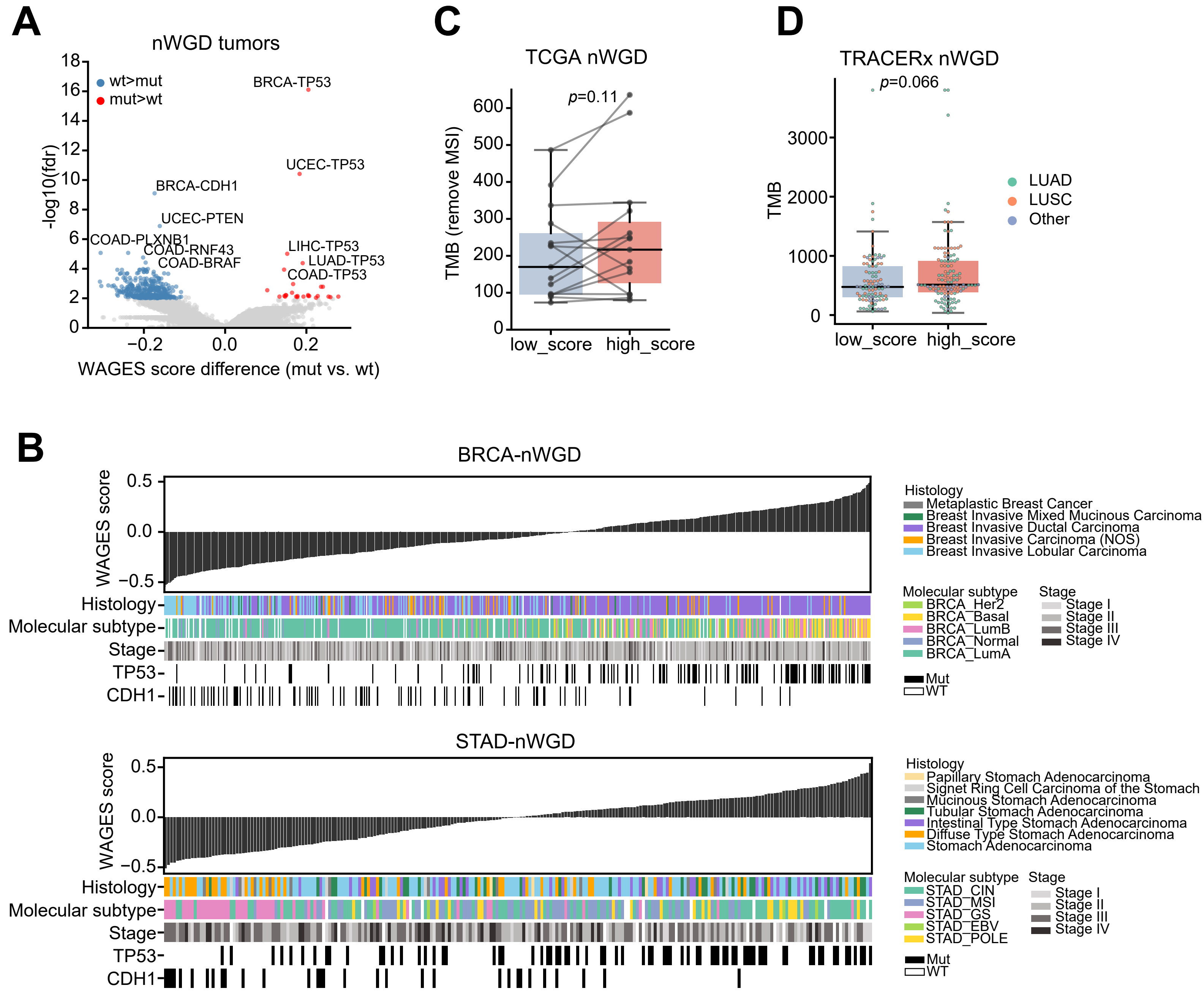

Fig. S6. Genomic correlates of WAGES scores. A, Association between WAGES score and gene mutation in TCGA nWGD tumors including MSI tumors. X axis, score difference between mutant and wildtype samples. Y axis, FDR at log10 scale. P values are calculated using two-sided Wilcox test and adjusted using the Benjamini-Hochberg method. **B**, Association of WGD signature and CDH1 in BRCA and STAD nWGD tumors is confounded by disease histology. The upper panel shows signature score in ascending order. The bottom panel shows histology, molecular subtype, tumor stage, TP53 and CDH1 mutation status. **C-D**, Tumor Mutation Burden (TMB) in low-score and high-score nWGD tumors in TCGA (C) and TRACERx lung (D). We excluded MSI tumors from the analysis. Each dot in TCGA data represents a cancer type stratified by signature score median. P values are calculated using paired t-test. For TRACERx lung, each dot represents a sample, and p values are calculated using two-sided Wilcox test.

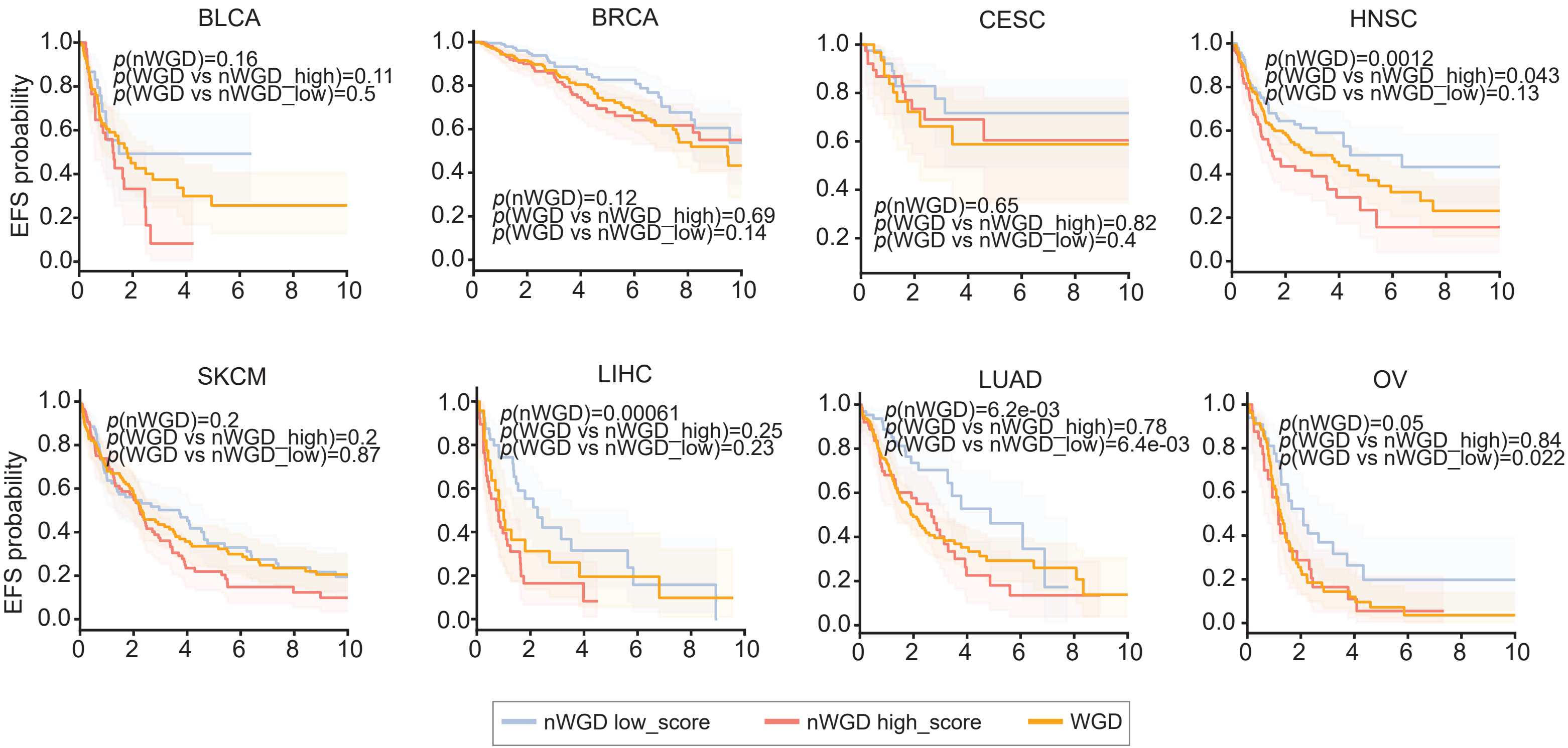

Fig. S7. Survival pattern of patients with high-WAGES nWGD tumors is comparable to WGD tumors. Kaplan-Meier curves for high- and low-WAGES score nWGD tumors. High-score and low-score nWGD tumors are split by median WAGES score for each cancer type. *P* values are calculated using log-rank test. *p*(nWGD) represents the log-rank *p* value between nWGD high_score and nWGD low_score groups. WGD samples are included for comparison.

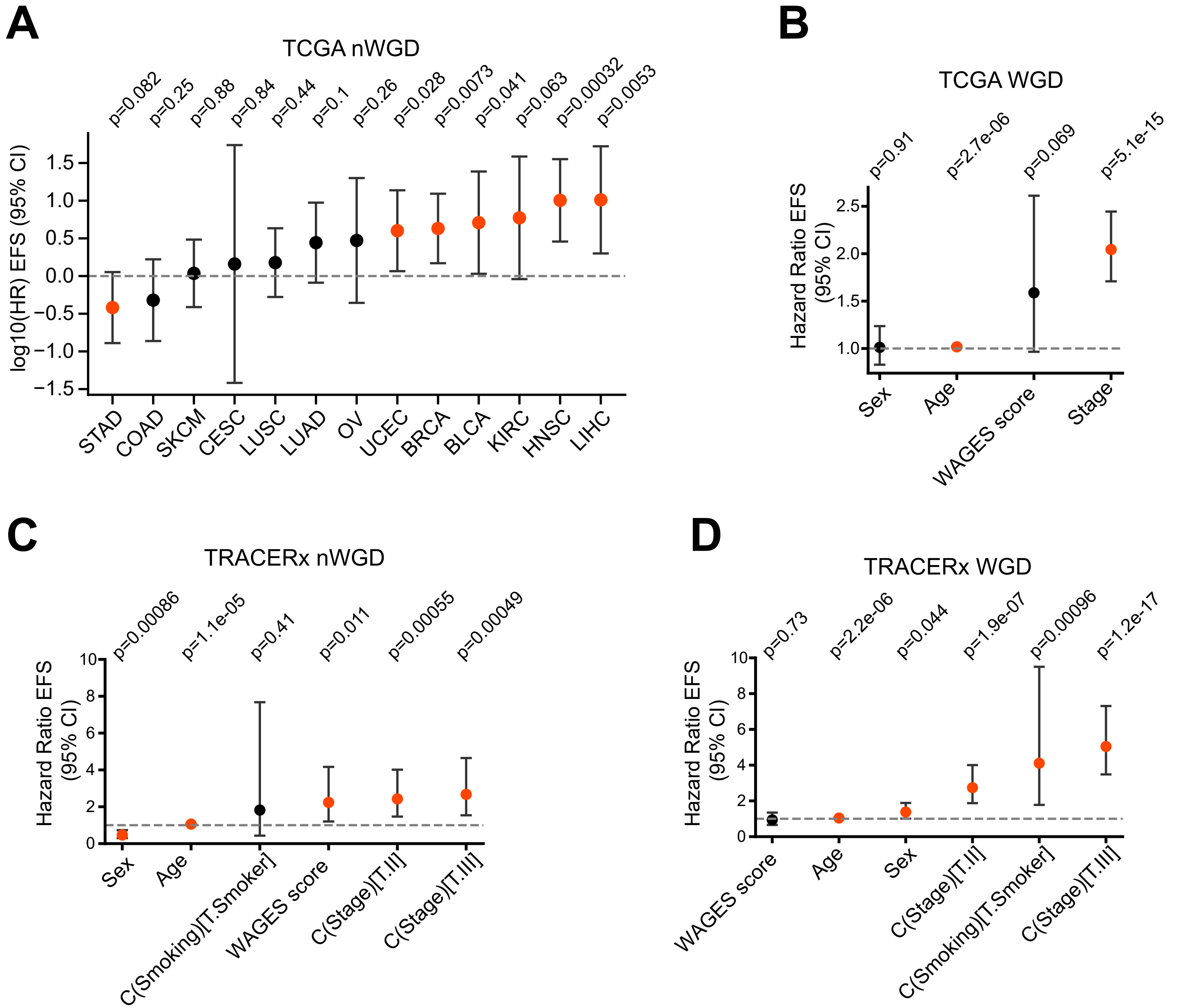

Fig. S8. WAGES score is correlated with patient prognosis in nWGD tumors. **A**, Cancer type specific hazard ratio of the WGD signature in TCGA nWGD samples after adjusting for tumor stage, patient age and sex. **B**, Similar to a but in WGD tumors. Cancer type is controlled as a variable. **C-D**, Hazard ratio of the signature in TRACERx lung nWGD (C) and WGD tumors (D) after adjusting for cancer type, tumor stage, patient age, sex and smoking status. *P* values in these figures are calculated using multivariable Cox proportional hazards regression, and red indicates statistical significance ($p<0.05$).

Table S1. (separate file)
WAGES gene list. The 598 genes constituting the WGD-Associated Gene Expression Signature (WAGES), derived from multivariable linear regression of gene expression.
Table S2. (separate file)
WAGES scores of TCGA samples. WAGES scores of 11,069 TCGA RNA sequencing samples.
Table S3. (separate file)
Genes associated with WAGES scores in nWGD tumors (after excluding MSI tumors). The 29 significant gene-cancer type pairs were identified by comparing WAGES scores between mutant and wildtype nWGD tumors.
Table S4. (separate file)
GSEA result for nWGD high-score versus low-score tumors.
Table S5. (separate file)
Longitudinal datasets used in the study. The eight published longitudinal cohorts with paired pre- and on- or post-treatment samples used to relate WAGES residuals to treatment response.
Table S6 (separate file)
Multivariable Cox proportional hazards model associates WAGES with patient event-free survival.
